# Elusive Gastric Atrophy in Children: Diagnostic Pitfalls and a Stepwise Approach

**DOI:** 10.1155/crpe/9929872

**Published:** 2026-05-28

**Authors:** Nikita Rodenbach, Annette Venter, Alfonso Rodriguez-Herrera, Julia Duder, Eimear Lee

**Affiliations:** ^1^ Paediatric Department, St Luke’s Hospital Kilkenny, Kilkenny, Co Kilkenny, Ireland; ^2^ Paediatric Department, University Hospital of Waterford, Waterford City, Co Waterford, Ireland; ^3^ Histopathology Department, University Hospital Waterford, Waterford City, Co Waterford, Ireland, hse.ie

## Abstract

Atrophic gastritis is an autoimmune gastritis that is characterised by the production of antibodies to parietal cells and intrinsic factor of the stomach. It is uncommon in paediatrics, as a result, is not usually in the differential diagnosis. A boy in middle childhood presented with a history of auditory hallucinations and fatigue, his workup only showed iron deficiency anaemia which responded to oral iron replacement but rebounded off therapy. There was no underlying cause found until years later, when he presented with Raynaud’s phenomenon leading to an autoimmune workup being done which showed him being weakly antinuclear antibody (ANA) positive and strongly positive for antiparietal cell antibody. This led to the patient undergoing endoscopy where the histopathology findings revealed antral‐like gastric mucosa with mild mixed chronic inflammation. There was a decrease in parietal cell numbers and t focal evidence of intestinal metaplasia, but no evidence of dysplasia or malignancy. This confirmed the diagnosis of atrophic gastritis which is extremely rare in the paediatric population.

**Learning Points/Take Home Messages**
• As a clinician, it is important to have an open mind when evaluating a patient especially when your workup has not provided answers as you had hoped. We need to understand our thought process and know how to minimise error.
• These patients do not always present with obvious signs and symptoms, and one should be on high alert if a patient has an autoimmune condition with a history of iron deficiency anaemia.
• There is a lack of evidence‐based guidelines, which will need to be developed for the management, follow‐up and especially screening for further autoimmune diseases in children with atrophic gastritis.

## 1. Background

Atrophic gastritis is an autoimmune gastritis that is characterised by the production of antibodies to parietal cells and intrinsic factor of the stomach. This leads to the destruction of the mucosa and increases the risk of malignancy development. It is also thought to increase the risk of other autoimmune conditions such as insulin‐dependent diabetes and thyroid disease [1].

This case has highlighted the fact that atrophic gastritis is uncommon in the paediatric populations, as a result, is not usually in the differential diagnosis. It shows us that some patients will only present with nonspecific symptoms such as iron deficiency anaemia or fatigue and will not always have the associated autoimmune comorbidities as is usually seen in these patients.

## 2. Case Presentation

A boy in middle childhood was referred to the Paediatric Outpatient Department with a short history of auditory hallucinations and nightmares. There was reported fatigue, but there were no complaints of abdominal pain, nausea, vomiting or diarrhoea. The father was noted to have hypothyroidism, and his mother had hyperthyroidism which required a thyroidectomy. His systemic examination was normal, and only pallor was noted. He was referred to the Community Adolescent Mental Health Services, and baseline bloods were done, namely, a full blood count, electrolytes and thyroid function.

On review of the results, he had a microcytic anaemia with a haemoglobin level of 7.4 g/dL (Reference 11.5–15.5). He was initiated on oral iron replacement with follow‐up investigations arranged in 4 weeks. His haemoglobin responded well and went up to 12.1 g/dL (Reference 11.5–15.5). His immunoglobulin levels were normal, and his antitissue transglutaminase was not suggestive of coeliac disease. There was no evidence of haemolysis with normal liver functions, negative direct Coombs test (DCT) and no antibodies (AB) were present. A low ferritin of 23 μg/L (Reference 47–358) was present. The blood film was analysed and reported as a microcytic anaemia suggestive of iron deficiency. The oral iron replacement continued, and follow‐up bloods arranged in 6 months’ time.

Over the subsequent 2 years, there appeared to be a response to oral iron replacement, but iron deficiency would reoccur once off therapy. The trend can be seen in Table 1. Other sources of blood loss were excluded by a negative faecal occult blood, negative urine dipstick and a negative calprotectin.

**TABLE 1:** A table representing the full blood count results over time.

Test	Units	Initial presentation	Two months later	Six months later	Two years 4 month later	Two years 7 months later
WBC	× 10^9/L	4.8	5.6	7.3	4.0	4.6
Hb	g/dL	**7.4 ↓**	12.1	13.5	**7.7 ↓**	14.4
MCV	Fl	**56.8↓**	**69.6 ↓**	82	**56.4 ↓**	**75.4 ↓**
MCH	Pg	**16 ↓**	**21.5 ↓**	28.7	**16 ↓**	**26.1 ↓**
MCHC	g/dL	**28.1 ↓**	**30.9 ↓**	35	**28.3 ↓**	34.6
Platelets	× 10^9/L	**416 ↑**	**405↑**	305	**401 ↑**	246

*Note:* Table created by the authors. All bold results are outside of the normal reference ranges as quoted by our laboratory.

The child re‐presented to our paediatric assessment unit years after the initial presentation with Raynaud’s phenomenon and nodular lesions on his fingers. Blood investigations, including an autoimmune screen, were done and a referral to rheumatology sent. The results revealed a weakly positive ANA, antiparietal cell Ab strong positive and a negative rheumatoid factor. His thyroid functions were normal. He had a normal haemoglobin, Vitamin B12, folate and ferritin. He was reviewed by a rheumatology specialist a few months later who diagnosed him with primary Raynaud’s and provided advice on conservative management. They advised a referral to a paediatric gastroenterologist for possible upper endoscopy due to the history of recurrent iron deficiency anaemia and new findings from autoimmune screen.

The gastroenterology referral led to the patient subsequently receiving a scope which was done under general anaesthesia and revealed atrophic gastritis on biopsy. Further investigations revealed high gastrin levels and persistent iron deficiency anaemia. He was once again initiated on oral iron replacement with continued monitoring planned.

Throughout this, he was reviewed by the mental health services and diagnosed with attention deficit disorder for which he was started on fluoxetine and methylphenidate hydrochloride.

### 2.1. Investigations

*Haematological workup*:

Blood film results: Elliptocytes 2+; dimorphic red cell picture; hypochromic microcytic; normochromic normocytic and reported as a microcytic anaemia suggestive of iron deficiency.

Direct Coombs negative.

No blood cell antibodies detected.

Reticulocyte count.

*Biochemical testing*:

Normal renal and liver function tests.

Normal thyroid functions on multiple occasions.

Normal immunoglobulin levels.

Normal vitamin B12, folate. Normal faecal calprotectin. Negative faecal occult blood. High Gastrin level 323 pg/mL (range < 126 pg/mL).

*Autoimmune workup*:

Antitissue transglutaminase antibodies negative.

ANA positive (weak).

Antiparietal cell AB positive (+4)

Antimitochondrial AB negative.

Antismooth muscle negative.

Antiliver/kidney microsomal AB negative.

Anti‐intrinsic Factor AB negative 2 U/mL (range negative < 7, equivocal 7–10, positive > 10 U/mL).

*Endoscopy results*:

As highlighted in the American Gastroenterological Association Clinical Practice Update on the Diagnosis and Management of Atrophic Gastritis, typical endoscopic features of atrophic gastritis include a pale appearance of the gastric mucosa, increased visibility of submucosal vasculature due to mucosal thinning, and loss or flattening of gastric folds. When intestinal metaplasia is present, additional findings such as light blue crests and white opaque fields may be observed. The document also emphasises that these mucosal changes are often subtle and may require optimised inspection techniques for adequate recognition [2].

**FIGURE 1:**
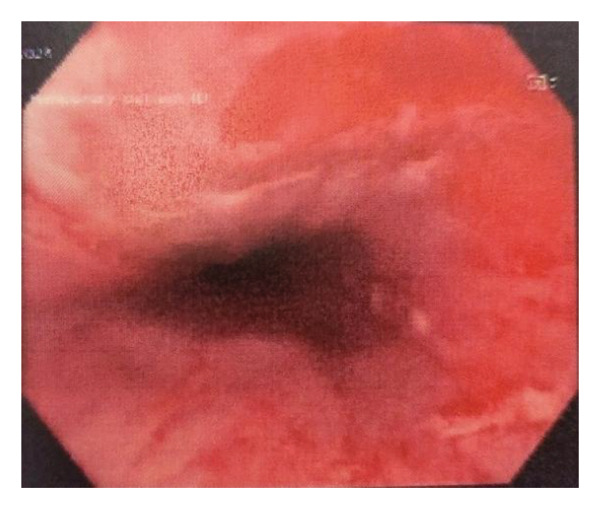
Endoscopic view of patients’ oesophagus. Photograph by the author.

**FIGURE 2:**
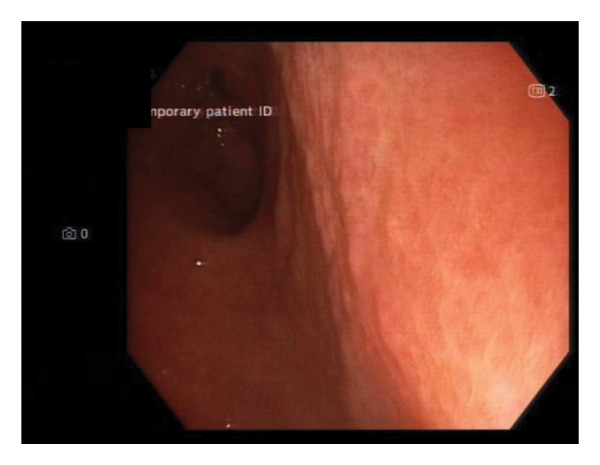
Stomach with minimal degree of fold flatness. Photograph by the author.

**FIGURE 3:**
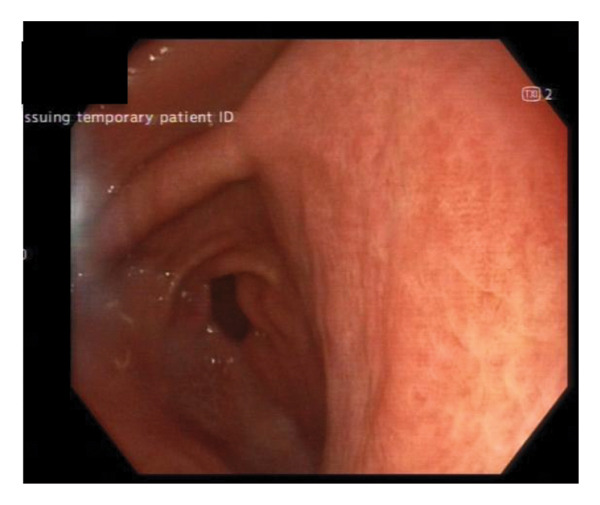
Stomach. No paleness of mucosa. No increased vascular appearance. Photograph by the author.

**FIGURE 4:**
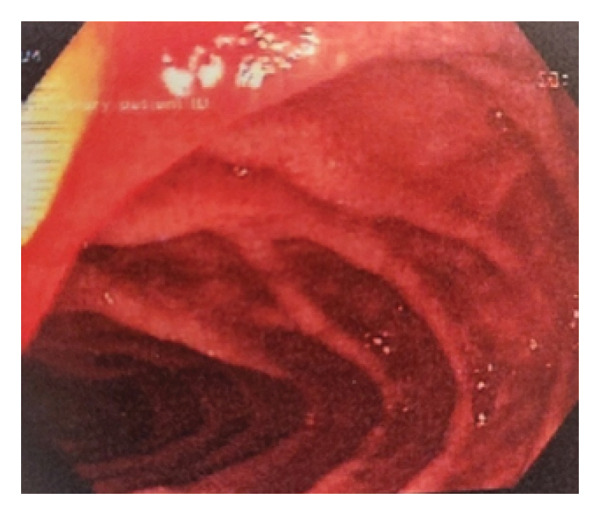
Endoscopic view of patients’ duodenum. Photograph by the author.

In the present case, upper gastrointestinal endoscopy did not demonstrate these classic macroscopic features. The gastric mucosa did not exhibit increased vascular pattern visibility. A minimal degree of fold flattening was appreciated. No light blue crests or white opaque fields were identified. Overall, the endoscopic appearance of both the antrum and corpus was considered macroscopically unremarkable (Figures 1, 2, 3, and 4).

This finding is consistent with the paediatric literature, where the most striking endoscopic feature of atrophic gastritis can be the absence of remarkable macroscopic abnormalities. Several reports describe children with normal‐appearing upper endoscopy in whom histological examination subsequently revealed chronic atrophic gastritis involving the fundic and/or antral mucosa. This highlights the limited sensitivity of white‐light endoscopy for detecting early or patchy atrophy in children [3].

*Histopathology*:

Regarding biopsy strategy, adult guidelines recommend systematic sampling (two antral biopsies, two corporal biopsies, and one from the incisura angularis) [4]. However, there is no established consensus on the optimal number and distribution of biopsies in the paediatric population. Importantly, paediatric studies have shown that atrophy may be detected predominantly in biopsies taken near the antrum–corpus junction, supporting the concept that atrophy can progress along an advancing antrum–corpus border (border zone). In our case, targeted biopsies included samples from both antral and corporal regions, allowing histological identification of corpus‐predominant atrophic changes despite the absence of characteristic endoscopic abnormalities.

**FIGURE 5:**
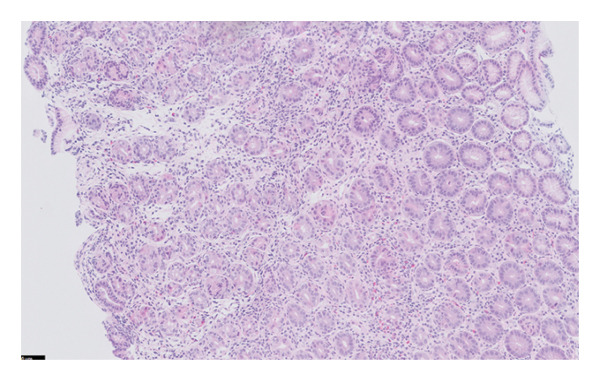
Histological features of autoimmune metaplastic atrophic gastritis include diffuse chronic inflammation, atrophy of oxyntic glands, intestinal metaplasia and enterochromaffin‐like cell (ECL) hyperplasia. It illustrates the loss of oxyntic cells and replacement by antral‐type mucosa in a background of chronic inflammation. Photograph by the author.

**FIGURE 6:**
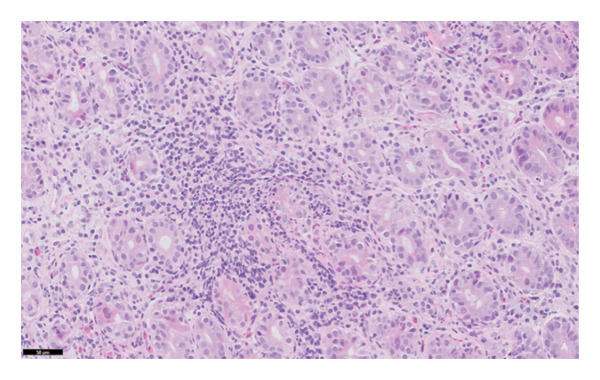
Higher power image highlighting the background of chronic inflammation. Photograph by the author.

**FIGURE 7:**
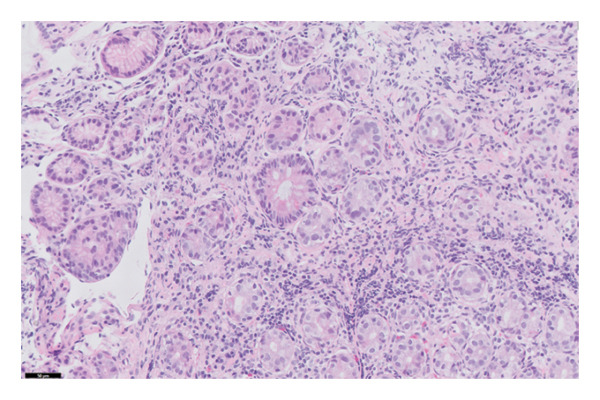
This image highlights a focus of intestinal metaplasia at the centre. Photograph by the author.

**FIGURE 8:**
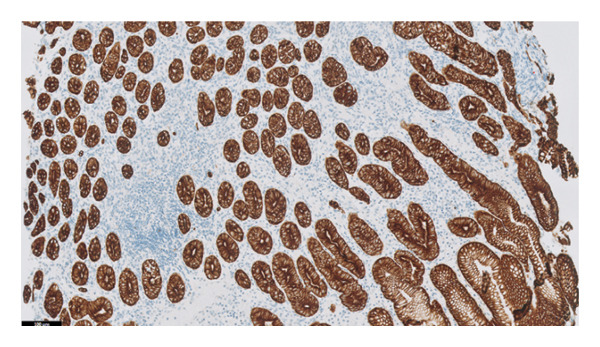
Cytokeratin stain AE1AE3 further highlights atrophy of glands. Photograph by the author.

**FIGURE 9:**
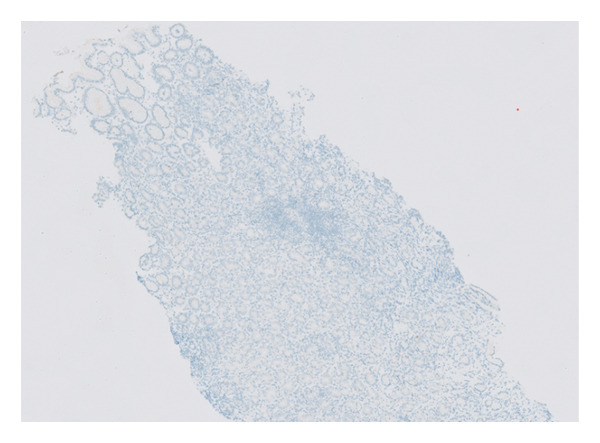
Gastrin immunostain is negative, confirming the absence of gastrin‐producing G cells in this gastric fundic mucosa. Photograph by the author.

**FIGURE 10:**
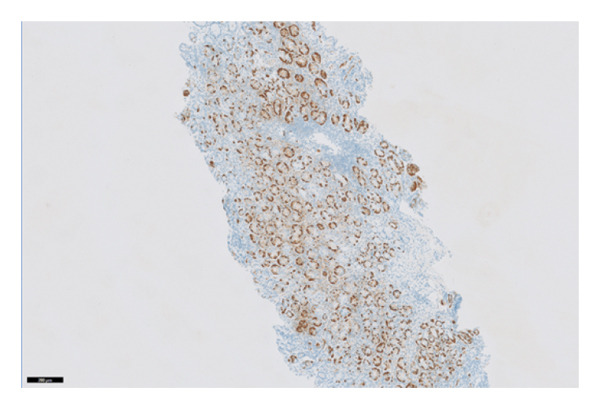
Chromogranin immunostain highlights enterochromaffin‐like cell (ECL) confirming ECL hyperplasia. In autoimmune metaplastic atrophic gastritis, there is a loss of parietal cells in the body of the stomach and therefore decreased acid production. This stimulates antral G cells to produce more gastrin, which stimulates ECL hyperplasia. Photograph by the author.

**FIGURE 11:**
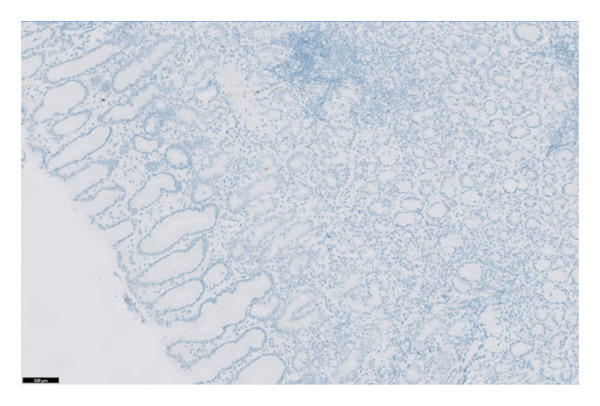
*Helicobacter pylori* organisms are not seen. Supported by negative immunostain for *H. pylori*. Photograph by the author.

The histopathology findings were as follows (Figures 5, 6, 7, 8, 9, 10, and 11):

Proximal, medial and distal oesophagus biopsy: Normal stratified squamous oesophageal mucosa. With no evidence of eosinophilic, oesophagitis or evidence of dysplasia or malignancy.

Stomach biopsy: Antral like gastric mucosa with mild mixed chronic inflammation composed of lymphocytes and eosinophils with small lymphoid aggregates. There is very focal acute inflammation. A gastrin stain is negative, confirming that the biopsy is from the body of the stomach. There is no significant decrease in gland density, but there are a decreased number of parietal cells. A chromogranin stain shows increased enterochromaffin‐like cells. There is focal evidence of intestinal metaplasia. Helicobacter‐like organisms are not seen, confirmed by immunohistochemistry. There is no evidence of dysplasia or malignancy. The findings are in keeping with early autoimmune metaplastic atrophic gastritis.

D2 biopsy: Small bowel mucosa with normal villous architecture. There is no increase in intraepithelial lymphocytes. No luminal parasites are seen. The appearance is of normal duodenal mucosa.

*Other*:

Urine dipstick negative for blood.

### 2.2. Differential Diagnosis

The commonest causes of iron deficiency anaemia in children would include poor intake with rapid growth, low birth weight and gastrointestinal loss from cow’s milk consumption [5]. The patient presented with a microcytic anaemia (most commonly due to iron deficiency anaemia) which is when the department started investigating the underlying cause.

We first ruled out a poor nutritional intake as his mother was insistent that the family had a good balanced diet in the home and he was eating a variety of iron rich foods. In older children who have a good oral intake, it is important to look for blood loss as the underlying cause [5]. Blood loss was investigated next and no occult blood in stool nor on urine dipstick was found. We then looked for possible malabsorption issues by looking for coeliac which was ruled out twice with antitissue transglutaminase antibodies being negative, and inflammatory bowel disease was ruled out with negative stool calprotectin. Pernicious anaemia was not present at this stage as there were normal B12 levels present. We tested for renal failure as a possible cause of the anaemia, and renal function was routinely checked and was always normal.

At this point, we had an iron deficiency anaemia without an underlying cause. The patient then presented with a rheumatological condition which prompted the further investigations to be done which included an autoimmune screen. The autoimmune studies came back normal except for weak ANA and strongly positive antiparietal cell antibody. This had led us believe that the child could possibly have had gastritis and arranged an endoscopy to conclude the diagnosis.

### 2.3. Treatment

There is no curative therapy for autoimmune atrophic gastritis so the focus will be on preventing vitamin B12 deficiency and iron deficiency [3]. The patient is receiving intermittent oral iron replacement as required based on his full blood counts with concurrent laxatives due to his development of constipation as a side‐effect. Dietetics input has been sought to increase his nutritional iron intake.

### 2.4. Outcome and Follow‐Up

The child will continue to follow up with the paediatric gastroenterologist who will continue to follow up his haemoglobin and iron levels to monitor the need for treatment.

Paediatric‐specific surveillance guidelines for autoimmune gastritis (AIG) are currently lacking and that follow‐up strategies in children are largely extrapolated from adult data or individualised according to clinical context [3]. We are arranging repeated endoscopy for identifying risk of malignancy every 2‐3 years at this point.

## 3. Discussion

Atrophic gastritis is an autoimmune inflammatory condition of the stomach, typically the corpus that is characterised by the production of antibodies (CD4+ T‐cell lead) to the proton pump H+/K+ adenosine triphosphatase (present in the gastric parietal cells) and to a lesser extent the intrinsic factor, leading to mucosal destruction and an increased risk of malignancy development which can cause unexplained iron and or Vitamin B12 deficiency. It is also thought to increase the risk of other autoimmune conditions such as insulin‐dependent diabetes and thyroid disease. The prevalence in the paediatric population is estimated to be much lower than the adult population, a retrospective study published in 2021 estimated the paediatric prevalence to be 0.15% compared to adults (2%–4%) [1, 6–8].

The presenting complaints can be vague and nonspecific, they can be related to nutritional deficiencies or can be neuropsychiatric as seen in our index case. A study carried out by Granat et al. published in 2024 quoted iron deficiency anaemia as the presenting complaint in 75% of patients. An observational study of children with confirmed autoimmune gastritis which included 3 girls and 2 boys highlighted that autoimmune gastritis should be considered as a diagnosis in children with refractory iron deficiency especially in the context of a family history of autoimmunity. In this same study, they concluded that children are more likely to present with iron deficiency anaemia than the typical presentation of pernicious anaemia (Vitamin B12 deficiency and megaloblastic anaemia) in adults. Refractory unexplained iron deficiency anaemia without gastrointestinal symptoms has also been found in adults (20%–27%). The three children in this study that had Vitamin B12 levels done had normal results. An interesting finding in this observational study of these 5 paediatric patients was that three families had a history of autoimmune disease—two thyroiditis and one with Type 1 diabetes mellitus. One patient had Type 1 diabetes and one was antitransglutaminase antibody positive. Parietal cell antibodies were positive in all five of these patients with none having autoantibodies for intrinsic factors which the authors attributed to positivity for an intrinsic factor increasing with age of patient and duration of illness [7, 9, 10].

In addition to the autoimmune comorbidities, a multicentre cohort study with 51 patients found an association with T helper 2 disorders: allergic rhinitis (7.8%), atopic dermatitis (9.8%), asthma (5.9%), eosinophilic oesophagitis (3.9%), and eosinophilic gastritis (9.8%) [11].

With regards to the investigations to help diagnose autoimmune gastritis, the following modalities can be used. Parietal cell and intrinsic factor antibodies can be tested with the former being the one that will more likely to be positive early in disease. Due to the rarity of this disease in paediatric patients, the sensitivity and specificity have not been established but in adults, parietal cell antibodies were quoted as being positive in 86.3% and in 0.47% for intrinsic factors alone and 13.2% for both. Other serum biomarkers that can be used are gastrin, Pepsinogen 1 and 2 and Ghrelin. Regular screening in established disease should be done to monitor for iron and Vitamin B12 deficiency as well as screening for associated autoimmune diseases: thyroiditis and Type 1 diabetes as there are strong links between autoimmune thyroiditis and Type 1 diabetes mellitus amongst those with atrophic gastritis. This was proven in a retrospective study where 59% of the 22 patients identified had an extra‐gastric immunological disorder. Another more recent multicentre retrospective study carried out by Granat et al. revealed autoimmune comorbidities in 62% of patients with positive serology versus 18% in serology‐negative patients [6, 9, 10].

This is not a condition that is diagnosed simply on blood investigations or simple bedside tests, and a biopsy is warranted even though it is an invasive procedure. Currently, there is no diagnostic criteria for the diagnosis of autoimmune gastritis. Endoscopic changes include gastric mucosal thinning, pallor with prominent blood vessels and softening of the folds of the stomach. During the endoscopy, the updated Sydney System is suggested which recommends two antral biopsy samples, one sample from the incisura angularis and two samples from cranially to the pyloric oxyntic border. Histologically, there is a loss of the gastric glandular structures in the oxyntic mucosa, which are replaced by antral glands leading to hypo/achlorhydria (which impairs iron absorption), low serum Pepsinogen 1 and hypergastrinemia. The hypergastrinemia leads to the proliferation of enterochromaffin‐like cells (ECL) which would be highlighted using a chromogranin immunostain [7, 12–14].

Management in children is primarily directed at correcting nutritional deficiencies, particularly iron and Vitamin B12, through oral or parenteral supplementation according to tolerance and response. Current evidence does not support reversal of histological atrophy with available therapies, and the disease is generally considered chronic and potentially progressive. In adult studies, proton pump inhibitors have been found to theoretically worsen the ECL hyperplasia, and as a result, should be discouraged. No immunosuppressants or biological agents are available for treatment due to a lack of evidence [7, 12].

A retrospective study was carried out from 2021 to 2022 to look at the clinical course and outcome of atrophic gastritis in children. They concluded that there was a high rate of metaplasia, and as such, surveillance is important, one study recommended five yearly endoscopic surveillance. The European Society of Gastrointestinal Endoscopy suggests surveillance every 3–5 years in adults with advanced atrophic gastritis, although the quality of evidence is limited. In the absence of paediatric‐specific data, current practice in children necessarily extrapolates from adult recommendations while individualising decisions according to histological severity, the presence of intestinal metaplasia, serological profile, family history and clinical evolution. This condition in children is becoming more diagnosed due to increased awareness and as a result guidelines should be developed to guide clinicians. We need to be aware that this is a preneoplastic condition especially with the long life expectancy of this patient group [3, 7].

As the above discussion highlights, this disease is a difficult one to identify in children and will require a workup that is thorough and excludes all the common causes. When our questions are not answered as expected by the usual explanations, practitioners need to be open to change their way of thinking. When a person has a mind that would only look for the prevalent and not be open to the exceptions, it could lead to the so called “diagnostic dilemma” with a resulting diagnostic delay for patients. A study by Lenti et al. quotes a diagnostic delay of 14 months in an adult population study of 291 patients, one can only imagine the delay in the paediatric population when it is rarely considered in the paediatric population. There was an article titled “The Thinking Doctor: Clinical decision‐making in contemporary medicine” which highlighted three main errors of thinking. What we learn from it is that we tend to follow a habitual course of action in a busy work environment and tend to follow general rules without looking outward [15, 16].

## Funding

No funding was received for this manuscript.

## Disclosure

No support from any organisation was received for the submitted work; no financial relationships with any organisations that might have an interest in the submitted work in the previous 3 years; there are no other relationships or activities that could appear to have influenced the submitted work.

## Consent

The patient as well as the patient’s father, due to the child being under 18 years old, had given consent for the case report relating to the patient’s medical condition and journey to be written up and published. They are aware and given consent for the photographs displayed. They are aware that they had the right to evoke their consent any time before publication.

## Conflicts of Interest

The authors declare no conflicts of interest.

## Patient Perspective

From the patient’s/parents’ perspective, the mothers’ intuition, instincts and persistence to request additional blood screening and referral to rheumatology were contributing factors in the successful diagnosis of both atrophic gastritis and Raynaud’s disease in 2023.
